# The Effects of Transcranial Direct Current Stimulation (tDCS) on Balance Control in Older Adults: A Systematic Review and Meta-Analysis

**DOI:** 10.3389/fnagi.2020.00275

**Published:** 2020-09-11

**Authors:** Zhenxiang Guo, Dapeng Bao, Brad Manor, Junhong Zhou

**Affiliations:** ^1^Sports Coaching College, Beijing Sport University, Bejing, China; ^2^China Institute of Sport and Health Science, Beijing Sport University, Bejing, China; ^3^Hebrew SeniorLife Hinda and Arthur Marcus Institute for Aging Research, Harvard Medical School, Boston, MA, United States

**Keywords:** tDCS—transcranial direct current stimulation, balance control, older adults, systematic review, meta-analysis

## Abstract

**Background:** Recently, considerable research has been conducted to study the effects of transcranial direct current stimulation (tDCS) on balance control in older adults. We completed a comprehensive systematic review and meta-analysis to assess the efficacy of tDCS on balance control in this population.

**Methods:** A search strategy based on the PICOS principle was used to find the literatures in the databases of PubMed, EMBASE, EBSCO, Web of Science. The quality and risk of bias in the studies were independently assessed by two researchers.

**Results:** Ten studies were included in the systematic review. A meta-analysis was completed on six of these ten, with a total of 280 participants. As compared to sham (i.e., control), tDCS induced significant improvement with low heterogeneity in balance control in older adults. Specifically, tDCS induced large effects on the performance of the timed-up-and-go test, the Berg balance scale, and standing postural sway (e.g., sway area) and gait (e.g., walking speed) in dual task conditions (standardized mean differences (SMDs) = −0.99~3.41 95% confidence limits (CL): −1.52~4.50, *p* < 0.006, *I*^2^ < 52%). Moderate-to-large effects of tDCS were also observed in the standing posture on a static or movable platform (SMDs = 0.37~1.12 95%CL: −0.09~1.62, *p* < 0.03, *I*^2^ < 62%).

**Conclusion:** Our analysis indicates that tDCS holds promise to promote balance in older adults. These results warrant future studies of larger sample size and rigorous study design and results report, as well as specific research to establish the relationship between the parameter of tDCS and the extent of tDCS-induced improvement in balance control in older adults.

## Introduction

Standing and walking are critical to most activities of everyday living. In older adults, diminished balance when standing and walking is a predictor to increased fall risk, loss of functional independence, morbidity, and mortality. In addition to spinal circuits and the peripheral neuromuscular system—which have been the primary foci of most traditional therapeutic efforts—the regulation of balance is also dependent upon numerous sensory, motor, and cognitive brain networks that enable selective attention to appropriate aspects of the visual field, the integration of numerous sensory inputs, selection of the appropriate walking speed, the ability to stand while performing necessary cognitive tasks, and numerous other functions that enable one to perform complex texts and navigate ever-changing environments (Lajoie et al., 1996; Yogev-Seligmann et al., 2008; Mirelman et al., 2014; Wollesen et al., 2016; Zhou et al., 2019). Aging and age-related diseases including Parkinson's disease and dementia are associated with both anatomical and biophysiological changes (Jové et al., 2014; Chu et al., 2019) that interact with aging and further alter brain network function (Chun et al., 2000; Benedetti et al., 2006; Beheshti et al., 2020) and balance control (Mahncke et al., 2006; Fjell et al., 2009). Strategies targeting these supraspinal elements of balance control therefore hold great promise to improve balance, safety, and independence within vulnerable older adult populations.

One promising strategy to modulate brain network function and in doing so the supraspinal control of balance in older adults is transcranial direct current stimulation (tDCS). tDCS safely and selectively modulates the excitability of brain networks (specifically the likelihood of neuronal firing) by sending low-level electrical currents between electrodes placed on the scalp (Nitsche and Paulus, 2000). This process generates an electric field that polarizes neuronal populations and modulates resting membrane potentials. The electric field generated by tDCS, and its effect on cortical excitability depend upon multiple factors including the tDCS montage (i.e., electrode type, size, polarity, and placement, current intensity), individual head and brain anatomy, and the conductivity of the involved tissues.

tDCS has been demonstrated to modulate cortical excitability in the aging brain (Summers et al., 2016) and induce functional improvements to numerous elements of the complex balance control system, including somatosensory function (Ragert et al., 2008), attention (Coffman et al., 2014), and reaction time (Fregni et al., 2006). Recently, researchers have also started to directly test the effects of tDCS on balance control in older adults and in those with neurodegenerative disease. Manor et al. (2015), for example, demonstrated that in healthy older adults, one session of tDCS designed to target the left dorsal lateral prefrontal cortex (dlPFC) improved standing and walking performance (e.g., reduced standing postural sway speed and increased walking speed) particularly when participants were standing and performing a concurrent cognitive task (i.e., dual tasking). Studies have also suggested that tDCS may improve balance control in individuals (though not necessarily older adults) suffering from movement disorders including chronic stroke (Zandvliet et al., 2018) and Parkinson's disease (Broeder et al., 2015; Swank et al., 2016; Dagan et al., 2018).

While the above research has supported tDCS as a potential strategy to balance control in older adults, large inter-subject and between-study variance in the effects of tDCS on both cortical activation and functional performance has been observed (Horvath et al., 2014; Laakso et al., 2015). The purpose of this study was to therefore to complete a systematic review and meta-analysis to quantitatively analyze the effects of tDCS on the performance of balance control specifically in older adults based upon available peer-reviewed publications to date, with the intent to highlight recent efforts, advances, and needed areas of future research in this important area.

## Methods

### Design

This systematic review and meta-analysis was conducted in accordance with the Preferred Reporting Items for Systematic Reviews and Meta-Analyses (PRISMA) (Higgins et al., 2003).

### Literature Search

We searched the following electronic bibliographic databases: PubMed, EMBASE, EBSCO, and Web of Science. We reviewed publications from January 2000 to February 2020. Searches were limited to English language publications only and no date restrictions were applied. The PICOS (Population, Intervention, Comparison, Outcome, and Study design) framework was used to develop and refine the search strategy. The following search terms were used to identify relevant literature in the databases: (“non-invasive brain stimulation” OR “Transcranial Electrical Stimulation” OR “Transcranial current stimulation” OR “Transcranial direct current stimulation” OR “tDCS” OR “neuromodulation”) AND (“balance” OR “mobility” OR “standing” OR “walking” OR “ambulation” OR “postural sway” OR “gait”) AND (“older adults” OR “elderly” OR “elder adults”).

### Selection Criteria

Articles were included if they met the following criteria:

· the participants were of mean age ≥60 years;
· the intervention used in the study was tDCS;
· the outcome measure included metrics of balance, gait, or mobility;
· the design of study was randomized controlled trial or crossover-controlled.

Articles were excluded if the language was non-English or using an animal model. Reviews and conference articles were also excluded from the analysis.

### Data Extraction

The information pertaining to the methodological and technical aspects in the screened studies was independently reviewed by two researchers. Specifically, the information included trial design, number of participants, age, gender, experimental conditions, outcome measures, results, drop-out rate, duration (min/session) of intervention, the density of the tDCS-induced current, stimulation target, the placement, polarity and size of the electrodes, and tolerance/side/adverse effects of tDCS intervention. Only the information approved by both researchers was used in the following analyses.

### Quality Assessment

The quality and risk of bias assessment of each included study were independently assessed by those two researchers using the Cochrane Risk of Bias Tool (Higgins et al., 2019). This tool contains six domains in which each was classified as low, unclear or high risk of bias. Disagreements about the risk of bias assessments were resolved by consensus or by consulting a third researcher.

### Statistical Analysis

To determine the effect size of the intervention and control, standardized mean differences (SMDs) were calculated as the mean difference in the effect of tDCS vs. sham (i.e., control) divided by the pooled standard deviation. Cohen's *d* effect size (with a 95% confidence interval) was used to adjust for small sample sizes. Effect sizes were classified as trivial (<0.2), small (0.2–0.5), moderate (0.5–0.8), or large (>0.8) (Cohen, 1988). Meta-analysis was performed in Review Manager (RevMan version 5.3, Cochrane Collaboration, Oxford, UK) using the inverse variance method for included studies that compared the effects of tDCS and sham stimulation on balance-related outcomes in older adults. A random-effect model was used to conservatively estimate the pooled effect in anticipation of heterogeneity across individual studies due to differences in participant and intervention characteristics. Statistical heterogeneity was assessed using Cochran's Q and *I*^2^ statistics. The level of heterogeneity was interpreted according to guidelines from the Cochrane Collaboration: *I*^2^ values of 25, 50, and 75% correspond to low, moderate and high heterogeneity, respectively (Higgins et al., 2003). Statistical significance was set at *p* < 0.05.

## Results

**Figure 1:**
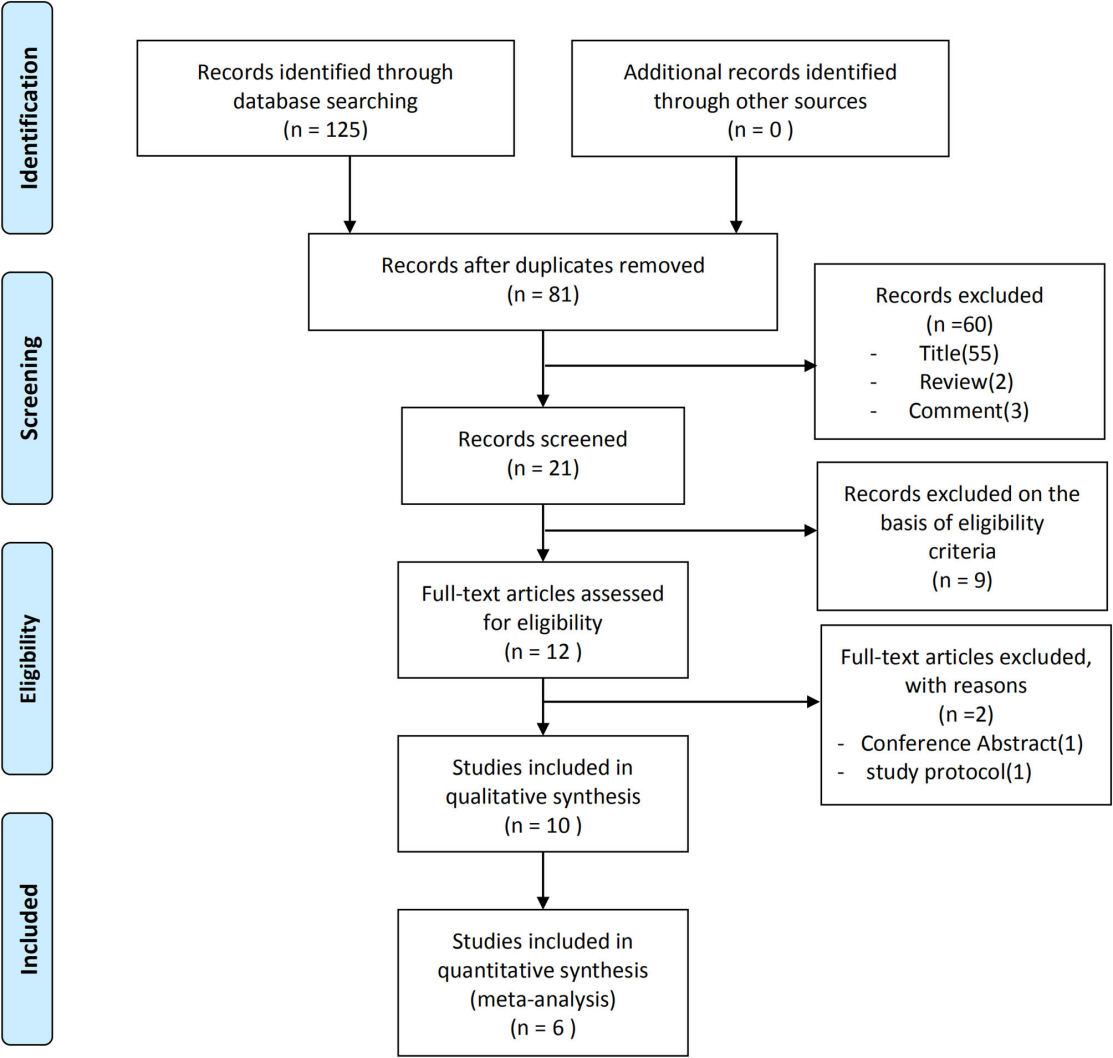
PRISMA flowchart of study selection.

The data assessment and analysis were completed on July 1st, 2020. After the removal of duplicates, the primary search identified 81 publication records (Figure 1). Following the inclusion/exclusion criteria, 12 studies were retained for full-text screening. After completing the full-text review, one conference abstract and one study without providing study protocol were excluded. Ten studies examining the effects of tDCS on balance and mobility in older adults with relatively healthy status or with functional impairment (e.g., stroke, slow gait) (Kaski et al., 2013; Manor et al., 2015, 2018; Saeys et al., 2015; Zhou et al., 2015, 2018; Ehsani et al., 2017; Kaminski et al., 2017; Nomura and Kirimoto, 2018; Yosephi et al., 2018) were accepted to be included in the systematic review and meta-analysis.

### Quality Assessment

**Figure 2:**
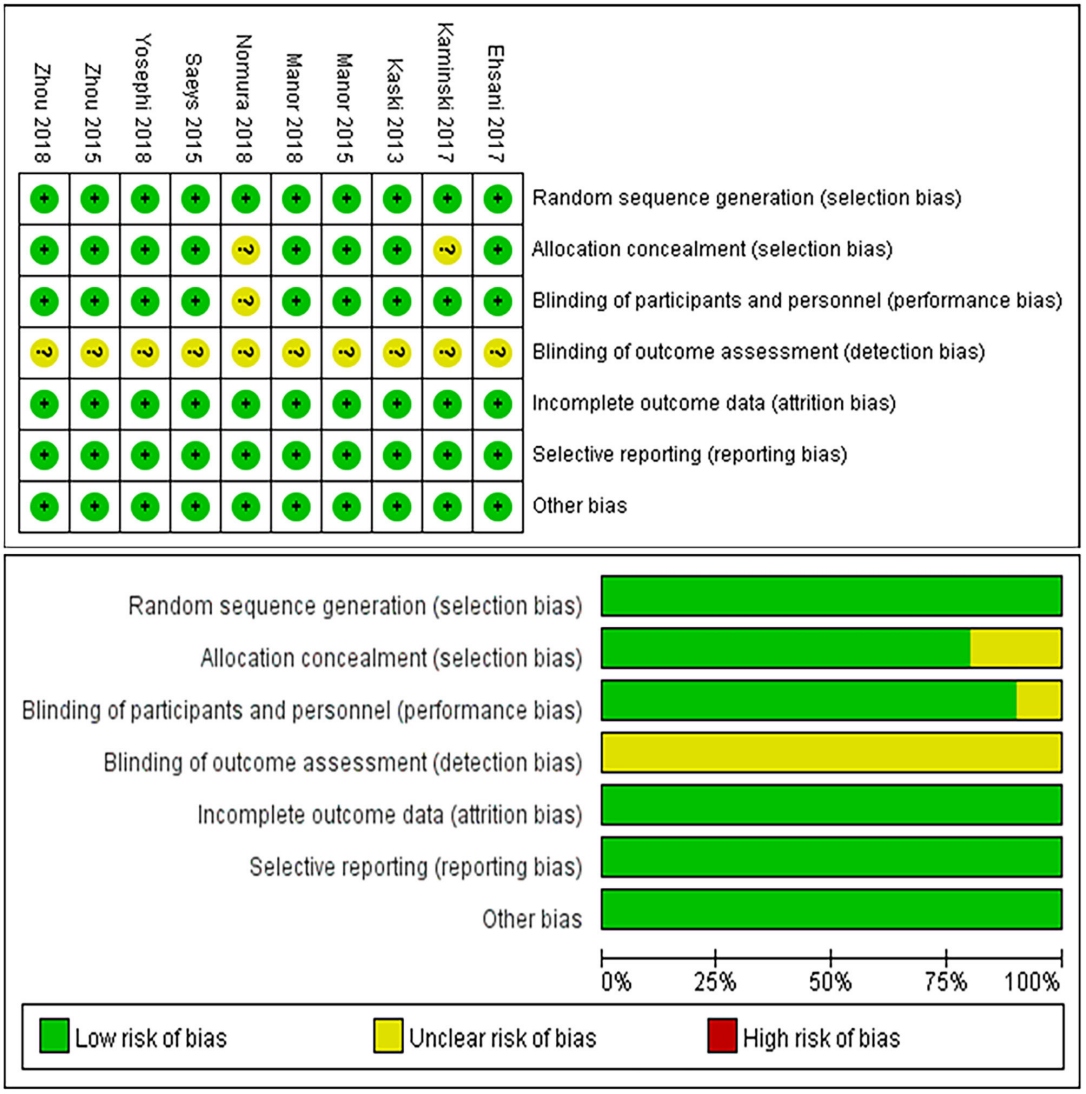
Results of Cochrane risk of bias tool.

Figure 2 illustrates the results of the Cochrane risk of bias tool. Eight studies implemented a double-blinded trial protocol (Kaski et al., 2013; Manor et al., 2015, 2018; Saeys et al., 2015; Zhou et al., 2015, 2018; Ehsani et al., 2017; Yosephi et al., 2018) and the other two studies were open-labeled (Kaminski et al., 2017; Nomura and Kirimoto, 2018).

### Participant Characteristics

Across all included studies, a total of 280 participants completed tasks assessing aspects of standing posture, gait, and/or mobility before and after either tDCS or sham stimulation, and the immediate and longer-term effects of tDCS on the task performance were examined. The sex of participants was reported in eight studies (Kaski et al., 2013; Manor et al., 2015, 2018; Saeys et al., 2015; Zhou et al., 2015; Ehsani et al., 2017; Kaminski et al., 2017; Nomura and Kirimoto, 2018), but not provided in the other two studies (Yosephi et al., 2018; Zhou et al., 2018). Participants were older adults without any overt neurological diseases, with history of stroke or suffering from Leukoaraiosis (Kaski et al., 2013; Saeys et al., 2015), or with slow gait (preferred gait speed <1.0 m/s) *and* cognitive executive dysfunction (Trial Making Test B performance below the 25th percentile of age- and education-matched normative values) (Manor et al., 2018). Details of the participant characteristics in each study are summarized in Table 1.

**Table 1:** Characteristics of participants in each study.

**Study**	**Total number**	**Age (years): mean ± SD**	**Sex**	**Health status**
Kaski et al. (2013)	9	79.4 ± 5.5	2F, 7M	Leukoaraiosis
Manor et al. (2015)	37	61 ± 5	25F, 12M	Healthy
Saeys et al. (2015)	31	63.22 ± 8.5	14F, 17M	Stroke
Zhou et al. (2015)	20	63 ± 3.6	9F, 11M	Healthy
Ehsani et al. (2017)	29	65.79 ± 6.11	16F, 14M	Healthy
Kaminski et al. (2017)	30	67.7 ± 6	17F, 13M	Healthy
Manor et al. (2018)	19	80.42 ± 4.18	10F, 9M	Slow gait and cognitive executive dysfunction
Nomura and Kirimoto (2018)	12	72.3 ± 5.3	8F, 4M	Healthy
Yosephi et al. (2018)	73	66.07 ± 4.37	N/A	High fall risk
Zhou et al. (2018)	20	61 ± 4	N/A	Healthy

*F, female; M, male; SD, standard deviation*.

### tDCS Characteristics

Table 2 shows the characteristics of tDCS intervention. tDCS montages with anodal electrodes placed over the targeted cortical region, with the goal of facilitating excitability within the targeted region, were used in all studies. All of them used sham stimulation as the control intervention. Seven of the studies used sponge electrodes of the same size (i.e., 35cm^2^) (Manor et al., 2015, 2018; Saeys et al., 2015; Zhou et al., 2015, 2018; Ehsani et al., 2017; Yosephi et al., 2018), while other three studies used electrodes of different sizes (Kaski et al., 2013; Kaminski et al., 2017; Nomura and Kirimoto, 2018). Kaminski et al. (2017) for example, used smaller-size anodes and larger-size cathodes.

**Table 2:** Characteristics of tDCS.

**Study**	**Main electrode**	**Electrode size (cm^**2**^)**	**Intensity (mA)**	**Current density (mA/cm^**2**^)**	**Duration (min)**	**Site of stimulation**	**Position of cathode**	**Number of sessions**
Kaski et al. (2013)	Anode	M = 40, R = 16	2	0.05	15	M1	Inion	1
Manor et al. (2015)	Anode	M = R = 35	2	0.06	20	Prefrontal regions	Right supraorbital region	1
Saeys et al. (2015)	Anode	M = R = 35	1.5	0.04	20	M1	Intact hemisphere	16
Zhou et al. (2015)	Anode	M = R = 35	2	0.06	20	Prefrontal regions	Right supraorbital region	1
Ehsani et al. (2017)	Anode	M = R = 25	1.5	0.06	20	Cerebellum	Right arm	1
Kaminski et al. (2017)	Anode	M = 25, R = 50	1	0.04	20	M1	Right frontal orbit	1
Manor et al. (2018)	Anode	M = R = 35	2	0.06	20	Prefrontal regions	Right supraorbital region	10
Nomura and Kirimoto (2018)	Anode	M = 9, R = 35	2	0.22	15	M1	Right supraorbital region	1
Yosephi et al. (2018)	Anode	M = R = 35	2	0.06	20	Cerebellum M1	Right buccinator muscle (Cerebellum tDCS); right supraorbital region (M1 tDCS)	6
Zhou et al. (2018)	Anode	M = R = 35	2	0.06	20	M1	Right supraorbital region	1

*tDCS, transcranial direct current stimulation; M, main electrode; R, reference electrode; M1, primary sensori-motor cortex*.

The target current intensity of tDCS used in these studies was set as 1, 1.5 or 2 mA (Table 2). In four studies, actual current was set at or below target depending upon participant comfort (Manor et al., 2015, 2018; Zhou et al., 2015, 2018). In these four studies, the current increased at the beginning of the intervention by 0.1 mA per second up to the maximum target current. However, participants were instructed to notify study personnel if the stimulation became uncomfortable. In these cases, stimulation intensity was set to 0.1 mA below the highest comfortable intensity level.

The duration of individual tDCS sessions was 20 min in the majority of studies (Manor et al., 2015, 2018; Saeys et al., 2015; Zhou et al., 2015, 2018; Ehsani et al., 2017; Kaminski et al., 2017; Yosephi et al., 2018). Two studies used tDCS sessions lasting 15 min (Kaski et al., 2013; Nomura and Kirimoto, 2018) (Table 2). Nine of ten studies implemented tDCS when participants were at resting state and the tasks were completed pre- and post-tDCS; while one study (Kaminski et al., 2017) implemented tDCS during the performance of task. Three studies explored the effects of multiple sessions of tDCS on the balance and mobility (Saeys et al., 2015; Manor et al., 2018; Yosephi et al., 2018), that is, participants completed the tests at baseline and within 3 days following the last session of intervention. Uniquely, in one study (Manor et al., 2018) the longer-term effects of tDCS was also examined by a follow-up assessment after 14 days following the last tDCS session. The other seven studies focused on the immediate after-effects of one session of tDCS (Kaski et al., 2013; Manor et al., 2015; Zhou et al., 2015, 2018; Ehsani et al., 2017; Kaminski et al., 2017; Nomura and Kirimoto, 2018), in which participants completed the tests immediately before and after one tDCS session. In two of these studies, participants repeated tests 15 min after the session, and again 48 h later (Ehsani et al., 2017; Nomura and Kirimoto, 2018). Three studies paired tDCS with another type of intervention, including physical training (Kaski et al., 2013), physical and occupational therapy (Saeys et al., 2015), and postural training (Yosephi et al., 2018).

Different cortical regions were targeted by tDCS, including: the cerebellum (Ehsani et al., 2017; Yosephi et al., 2018), the primary sensori-motor regions (Kaski et al., 2013; Saeys et al., 2015; Kaminski et al., 2017; Nomura and Kirimoto, 2018; Yosephi et al., 2018; Zhou et al., 2018), and the left dorsolateral prefrontal cortex (Manor et al., 2015, 2018; Zhou et al., 2015) (Table 2). Most included studies placed the cathode on the right supraorbital region. One study placed the cathode over the inion (Kaski et al., 2013), two studies targeting the cerebellum placed the cathode on the right arm and buccinator muscle (Ehsani et al., 2017; Yosephi et al., 2018). Another study focusing on patients with chronic brain damage due to stroke placed the cathode on the motor cortex of intact hemisphere with anodes placed on motor cortex of impaired hemisphere (Saeys et al., 2015).

### Study Outcomes

Balance was assessed by measuring standing postural sway (i.e., the center of pressure movement when standing on the force plate or balance system), gait metrics (e.g., gait speed) during walking, and/or performance of commonly-used dynamic balance tests, including the Tinetti test, the retropulsion test, time to maintain balance in a whole-body dynamic balancing task, Berg balance scale (BBS) and timed-up-and-go (TUG) test. Uniquely, three studies assessed standing and walking performance in single (e.g., standing or walking quietly with eyes open), and dual (e.g., standing or walking while performing a task serial subtraction by three from a random three-digit number) task conditions.

### The Effects of tDCS on Balance Control

Compared to sham, tDCS induced significant improvement in at least one aspect of balance in older adults in eight of ten studies (Table 3). One study reported only marginal effects of tDCS designed to target the primary sensory cortex on TUG performance in healthy older adults (Zhou et al., 2018). Another study observed no improvement in performance on a dynamic balancing test following tDCS as compared to sham (Kaminski et al., 2017).

**Table 3:** Characteristics of study outcomes.

**Study**	**Intervention**	**Measurement tool/outcome measure(s)**	**Measurement time**	**Results**	**Conclusion**	**Blinding efficacy**
Kaski et al. (2013)	tDCS + physical training	Six meter walk: speed, stride length, CoV of stride length; TUG: duration; Retropulsion test: duration to recovery	Before and immediately after one session of stimulation	Six meter walking: speed ↑, stride length ↓, CoV of stride length ↑; TUG: duration ↓; Retropulsion test: duration to recovery ↓;	Balance: ↑	N/A
Manor et al. (2015)	tDCS	Sixty seconds standing in ST and DT: sway speed, sway area; Fifty meter walking in ST and DT: walking speed;	Before and immediately after one session of stimulation	Standing: ST: sway area →, sway speed →; DT: sway area ↓, sway speed ↓; Walking: ST: walking speed →; DT: walking speed ↑;	Balance in ST: → Balance in DT: ↑	*p* = 0.29
Saeys et al. (2015)	tDCS	Tinetti test: total score;	Before and immediately after one session of stimulation	Tinetti test: total score ↑;	Balance: ↑	N/A
Zhou et al. (2015)	tDCS	Sixty seconds standing in ST and DT: sway complexity	Before and immediately after one session of stimulation	ST: sway complexity → DT: sway complexity ↑	Balance in ST: → Balance in DT: ↑	N/A
Ehsani et al. (2017)	tDCS	Dynamic and static balance test: APSI, MLSI, OSI; BBS: total score;	Before, immediately after, and 48 h after one session of stimulation	Dynamic and static balance: APSI ↑, MLSI ↑, OSI ↑; BBS: total score ↑	Balance: ↑	N/A
Kaminski et al. (2017)	tDCS	DBT: duration to maintain balance on a movable platform	During, and one day after the stimulation	Time to maintain balance: →	Balance: →	N/A
Manor et al. (2018)	tDCS	TUG: duration; Sixty seconds standing in ST and DT: sway speed, sway area; Fourteen foot walking in ST and DT: speed, stride time, CoV of stride time	Before and immediately after one session of stimulation, and after ten sessions of stimulation	TUG: duration → →; Standing: ST: sway speed → →, Sway area → →; DT: sway speed ↓↓, sway area ↓↓, dual task costs ↓↓; Walking: ST: all metrics → →; DT: speed →, stride time ↓, CoV of stride time ↓;	Balance in ST: → Balance in DT: ↑	*p* = 0.39
Nomura and Kirimoto (2018)	tDCS	AP and ML standing postural sway during arm upward task: sway speed, RMS, path length	Before, immediately after, and 15 min after stimulation	AP direction: sway velocity ↓↓, path length ↓↓, RMS → → ML direction: sway velocity → →, path length ↓↓, RMS → →	Balance: ↑	N/A
Yosephi et al. (2018)	tDCS + postural training;	Dynamic and static balance test: APSI, MLSI, OSI; BBS: Total score;	Before and after six sessions of stimulation	Dynamic and static balance: APSI ↑, MLSI ↑, OSI ↑; BBS: total score ↑	Balance: ↑	*p* = 0.98
Zhou et al. (2018)	tDCS	TUG: duration;	Before and immediately after one session of stimulation	TUG: duration →	Balance: →	*p* = 0.51

*tDCS, transcranial direct stimulation; ST, single task; DT, dual task; AP, anterioposterior; ML, mediolateral; TUG, timed up and go; APSI, anterior-posterior stability index; MLSI, medial-lateral stability index; OSI, overall stability index; BBS, berg balance scale; DBT, dynamic balancing task; RMS, root mean square*.

*↑: increase; ↓: decrease; → : no effect*.

Studies which implemented the dual task paradigm showed that tDCS targeting the prefrontal cortex induced significant improvement of standing postural control and gait in dual task condition in older adults, but no such effects were observed in single task, quiet standing conditions. Moreover, Manor et al. (2018) reported that 10 sessions of daily tDCS targeting the prefrontal region can induce longer-term improvement of balance and mobility in dual task conditions, which sustained at least 14 days following the last intervention.

### Blinding Efficacy of tDCS

Eight of 10 studies used double-blinded study design, and only four of them reported the blinding efficacy (Table 3). The reported blinding efficacy (*p* > 0.29) revealed successful blinding in these four studies.

### Side Effects and Attrition

The side effects of tDCS were not reported in three studies (Kaski et al., 2013; Saeys et al., 2015; Nomura and Kirimoto, 2018). Each study that reported side/adverse effects of tDCS observed that no adverse events were induced by tDCS (Manor et al., 2015, 2018; Zhou et al., 2015, 2018; Ehsani et al., 2017; Kaminski et al., 2017; Yosephi et al., 2018).

### Meta-Analysis

Multiple aspects of balance control were assessed across the ten studies included in the systematic review. Three studies uniquely measured balance by the Tinetti test score, standing postural sway complexity, or the time to maintain balance in a dynamic balance task (Saeys et al., 2015; Zhou et al., 2015; Kaminski et al., 2017). These three studies were thus not included in the following meta-analysis, since they may have increased the heterogeneity of results (Higgins et al., 2019). Another study was also not included in the meta-analysis because it did not report the mean and standard deviation of included balance outcomes and its authors did not respond to email query (Nomura and Kirimoto, 2018). We thus completed the meta-analysis on the remaining six studies, which tested the effects of tDCS as compared to sham stimulation on balance control in older adults without overt neurological disease.

### Effect of tDCS on Berg Balance Scale (BBS)

**Figure 3:**
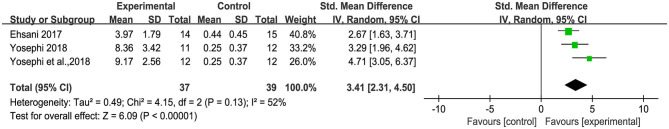
Forest plot of pooled and individual study effect sizes for Berg balance scale in tDCS vs. sham.

The overall pooled effect estimates from three comparisons (two studies) demonstrated that compared to sham, tDCS increased the BBS [SMD = 3.41 (2.31, 4.50), large effect] (Figure 3). Moderate statistical heterogeneity contributed to the imprecision in the estimate (*I*^2^ = 52%, χ^2^ = 4.15, *p* = 0.13). Sensitivity analysis showed that the study reported by Yosephi et al. (2018) had a much larger effect size than the other studies [SMD = 4.71 (3.05, 6.37)], inflating heterogeneity. When this individual effect estimate was removed from the meta-analysis, the *I*^2^ dropped from 52 to 0% without influence on the overall pooled effect. We thus included this study in the meta-analysis.

### Effect of tDCS on TUG

**Figure 4:**
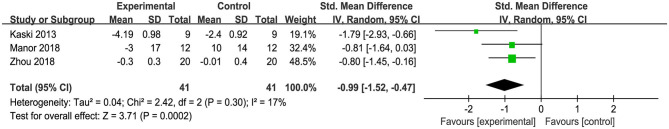
Forest plot of pooled and individual study effect sizes for TUG performance in tDCS vs. sham.

The effect of tDCS on the time to complete the TUG test of mobility was examined in three studies. An overall significant large effect size of −0.99 (95% CI: −1.52 to −0.47, *p* = 0.0002, Figure 4) was observed in tDCS compared to sham. Low heterogeneity was presented between studies (*I*^2^ = 17%, χ^2^ = 2.42, *p* = 0.30).

### Effect of tDCS on Static and Standing Balance Control

**Figure 5:**
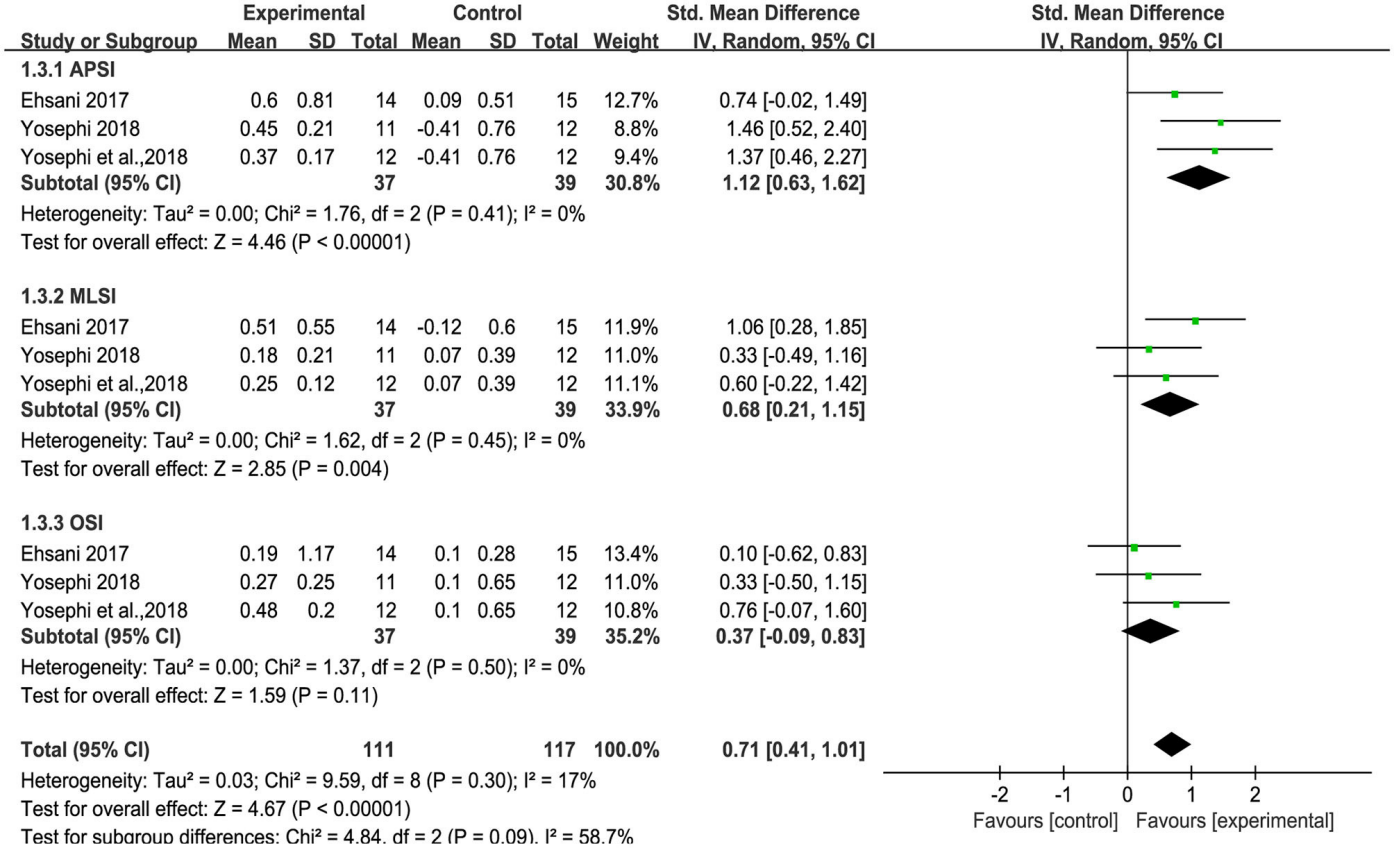
Forest plot of pooled and individual study effect sizes for static balance in tDCS vs. sham.

**Figure 6:**
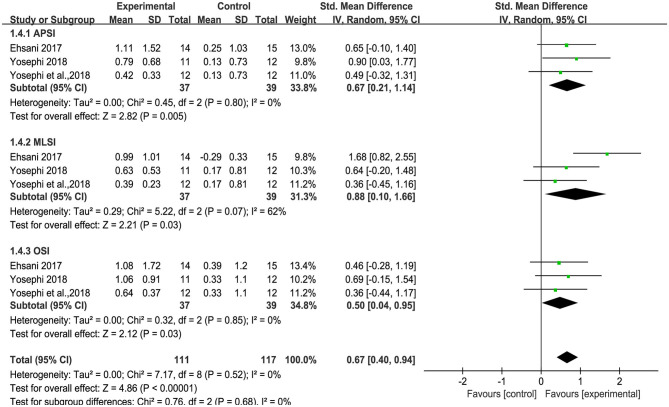
Forest plot of pooled and individual study effect sizes for dynamic balance in tDCS vs. sham.

Three studies used anterior-posterior stability index (APSI), medial-lateral stability index (MLSI) and overall stability index (OSI) to measure standing balance. An overall moderate to large significant effect size of 0.71 (95% CI: 0.41 to 1.01, *p* < 0.00001, Figure 5) of tDCS on APSI, MLSI and OSI in static balance control condition was observed compared to sham. Low heterogeneity was presented between studies (*I*^2^ = 17%, χ^2^ = 9.59, *p* = 0.30). Subgroup analysis showed that compared to sham, tDCS was associated with large effect size on APSI [SMD = 1.12 (0.63, 1.62), *p* < 0.00001], moderate effect size on MLSI [SMD = 0.68 (0.21, 1.15), *p* = 0.004], and a small, non-significant effect size on OSI [SMD = 0.37(−0.09, 0.83), *p* = 0.11]. An overall moderate to large effect size of 0.67 (95% CI: 0.40 to 0.94, *p* < 0.00001, Figure 6) of tDCS on APSI, MLSI and OSI in dynamic balance control condition was observed compared to sham. No heterogeneity was observed between studies (*I*^2^ = 0%, χ^2^ = 7.17, *p* = 0.52). Subgroup analysis examined the effects of tDCS on each outcome separately and showed that compared to sham, tDCS induced moderate significant effect size on APSI [SMD = 0.67 (0.21, 1.14), *p* = 0.005], large significant effect size on MLSI [SMD = 0.88 (0.10, 1.66), *p* = 0.03], moderate significant effect size on OSI [SMD = 0.50 (0.04, 0.95), *p* = 0.03].

### Effect of tDCS on Dual Task Standing and Walking Performance

**Figure 7:**
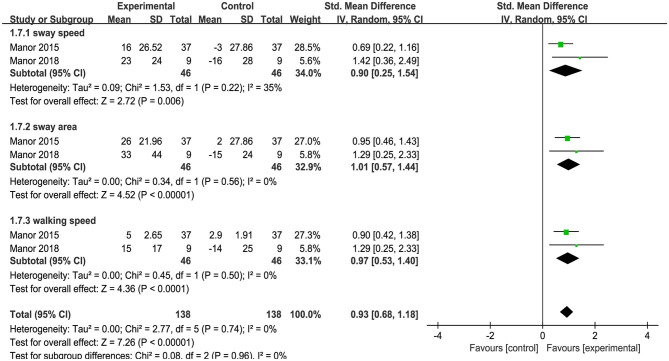
Forest plot of pooled and individual study effect sizes for dual task cost of standing and walking performance in tDCS vs. sham.

Uniquely, two studies provided information on the effects of tDCS on dual task balance performance, as measured by the dual task cost (i.e., the percent change of task performance from single to dual task condition) to metrics of standing postural sway or gait. The results indicated that as compared to sham, tDCS induced a large improvement of dual task standing and walking performance (i.e., less dual task cost) [SMD = 0.93 (0.68, 1.18), *p* < 0.00001, Figure 7]. No heterogeneity was presented between studies (*I*^2^ = 0%, χ^2^ = 2.77, *p* < 0.00001). Subgroup analysis examined the effects of tDCS on standing and walking performance separately, and showed that compared to sham, tDCS induced large significant effect size on sway speed [*I*^2^ = 35%, SMD = −0.90 (−1.54, −0.25), *p* = 0.006], sway area [*I*^2^ = 0%, SMD = −1.01 (−1.44, −0.57), *p* < 0.00001], and walking speed [*I*^2^ = 0%, SMD = 0.97 (0.53, 1.40), *p* < 0.0001].

## Discussion

This systematic review and meta-analysis indicate that tDCS has potential to promote balance control in older adults. Multiple aspects of balance were assessed within those studies included in the present analyses, and all but one study reported benefit to at least one balance-related metric. The majority of studies to date, however, have been small in sample size (i.e., number of participants was smaller than 30 in six studies) and focused on the immediate after-effects of a single exposure to stimulation. Considerable between-study variation in tDCS targets, electrode size and placement, current intensity, and duration exists. Moreover, important aspects of study results, blinding, and tDCS side effects were often not fully reported, resulting in a relatively small number of studies that could be included in the systematic review (i.e., ten studies) and even fewer in the meta-analysis (i.e., six of the ten studies). Larger, more definitive trials with rigorous methodology and form of results reporting are therefore needed to more fully understand the therapeutic potential of tDCS to improve balance in older adults.

Within the ten studies included in the systematic review, tDCS was designed to target several different brain regions (e.g., the cerebellum, primary sensori-motor cortex, or dorsolateral prefrontal cortex) and delivered using different current intensity (e.g., 1, 1.5, or 2 mA), size of electrodes (e.g., 35 or 25 cm^2^), stimulation duration (e.g., 15 or 20 min) and intervention duration (e.g., one session or multiple once-daily sessions). The positions of the cathode also varied considerably across studies. Furthermore, each of these studies included only one active intervention group, and each utilized an inactive “sham” stimulation protocol as the control. It thus remains unclear whether reported tDCS-induced benefits to balance stemmed from engagement of the specific cortical target and its connected neural networks, or from a more generalized facilitation of brain activity due to stimulation. Future studies that expose participants to multiple “active” tDCS interventions that each target a different brain region, and combine balance assessments with neurophysiologic recordings of target engagement and/or brain activation during balance tasks, are thus needed to more fully understand the neural mechanisms underlying observed tDCS balance improvements.

All studies included in this systematic review and meta-analysis utilized “bipolar” stimulation delivered via two relatively large sponge electrodes, with the anode placed on the scalp over the target brain region (and cathode placed over a non-target region of the brain or upper body). Such an approach induces a relatively diffuse electric field over the cortex that likely induces non-trivial effects in off-target regions (Ruffini et al., 2013). Moreover, the structure of the head and brain varies considerable across individuals. In older adults, such anatomic and functional variance in brain anatomy is often greater (Andrews-Hanna et al., 2007; Heuninckx et al., 2008; Raz et al., 2010). The same tDCS montage, therefore, may generate different electric fields at the level of the cortex across participants and result in significantly different on-target “dose” of tDCS. This issue is of particular importance because recent studies utilizing electric field modeling of brain MRIs to estimate current flow have demonstrated that the magnitude of the normal component of the electric field over the brain target correlated with the extent of the observed functional improvement; that is, participants who received greater “on-target” current dose performed better (Kim et al., 2014; Li et al., 2015). Kim et al. (2014), for example, demonstrated in a group of healthy younger adults that greater “on-target” current intensity correlated with the extent of tDCS-induced improvements in reaction time and verbal working memory. In future studies, researchers are thus encouraged to take advantage of modeling techniques to identify the most appropriate tDCS montage to stimulate a given brain region (Ruffini et al., 2013, 2014; Opitz et al., 2015), and if possible, perform such modeling on participant brain MRIs to individually-tailor such montages. Such approaches promise to advance the field by estimating and controlling the tDCS dose across individuals within a study.

Across the included studies, multiple different cortical targets were selected, all with the goal to improve balance. With the respect to balance control, multiple brain functional networks pertaining to motor control, attention, and cognitive function are indeed more or less involved (Zhou et al., 2019), and the importance of those regions in the control of balance may vary across age, disease states of population and the aspects of balance measured in the study (e.g., dynamic or static balance). One study included in our analysis (Kaminski et al., 2017) reported that compared to sham, tDCS designed to target the leg area of motor cortex did not improve the performance in the whole body dynamic balancing task in a sample of older adults; while the same researchers reported improvement in the same type of balance task in a group of younger adults using the same tDCS protocol (e.g., the same cortical targets) (Kaminski et al., 2016). One potential reason was argued that compared to younger cohort, the brain regions that were involved in completing the balancing task may be different in older adults. It is thus necessary in future work to appropriately identify and localize the stimulation targets for a given balance task in different populations, instead of roughly following the theoretical model or standard brain template (e.g., electroencephalogram (EEG) 10/20 system).

Nine of the ten studies implemented tDCS when participants were resting and not engaged in a particular task. Only one study implemented tDCS when participants were performing task (Kaminski et al., 2017). Previous studies in younger populations suggest that the effectiveness of tDCS may be influenced by the state of participants both before and during stimulation (i.e., state-dependency of tDCS), including the level of neural activation in the targeting brain regions, and functional performance of participants (Benwell et al., 2015; Learmonth et al., 2015; Nitsche and Bikson, 2017). The timing of tDCS exposure, as well as the between-subject variance in balance control performance at baseline, may thus impact the effects of tDCS on balance. Such factors, however, have not yet to tested within older adult populations.

Both aging and age-related diseases are associated with altered glutamatergic, GABAergic, cholinergic, and/or dopaminergic processes in the brain that have been linked to cognitive-motor (dys)function (Weng et al., 2018; Zou et al., 2018; Zhu et al., 2019). tDCS appears to modulate these neurobiological processes (Kuo et al., 2007; Stagg et al., 2011; Caumo et al., 2012; Filmer et al., 2014). Stagg et al. (2011), for example, demonstrated that one 10-min session of tDCS targeting M1 decreases GABA in this region in healthy younger adults, and that this decrease was associated with the improvement in reaction time. As no studies included in the meta-analysis focused specifically on individuals aged >60 years with neurological disease, future studies are needed to examine the neurophysiological and neurobiological effects of tDCS that may underlie improved balance control—both in older adults without overt disease and in those older adults suffering from neurodegenerative movement disorder.

Most studies included in this analysis focused on the immediate effects of a single session of tDCS. The longer-term effects of multi-session tDCS interventions on balance control in older adults remains unclear. Moreover, nine of the ten studies included in our systematic review employed a double-blinded design, yet only four reported blinding efficacy. It is thus strongly encouraged for such information to be reported in future studies, as similar sham interventions applied within neuropsychological studies have reported relatively poor blinding efficacy (Aslaksen et al., 2014; Xiao et al., 2020). As only six publications were included in the meta-analyses, we did not perform funnel plot analysis to assess the risk of bias (Higgins et al., 2019). It should also be noted that studies with positive results are more likely to be published. Additionally, the effect sizes generated by our meta-analysis may have been affected by the heterogeneity of balance-related outcome measures across included studies. Thus, while this systematic review and meta-analysis suggested that tDCS holds strong promise to improve balance in older adults (at least in those without overt disease), results should be treated with caution until larger, well-controlled trials are completed.

## Data Availability Statement

The raw data supporting the conclusions of this article will be made available by the authors, without undue reservation.

## Author Contributions

ZG contributed to literature search, figures, study design, data analysis, data interpretation, and writing. DB contributed to study design, data analysis, data interpretation, and writing. BM contributed to data interpretation and writing. JZ contributed to study design, figures, data analysis, data interpretation, and writing. All authors contributed to the article and approved the submitted version.

## Conflict of Interest

The authors declare that the research was conducted in the absence of any commercial or financial relationships that could be construed as a potential conflict of interest.
